# Supplementary material for: Analysis of the Effects of Beauveria bassiana Appressorium Formation on Insect Cuticle Metabolism Based on LC-MS
Source: J Fungi (Basel). 2025 Aug 15;11(8):595. doi: 10.3390/jof11080595 (PMC12387848; doi:10.3390/jof11080595)
Supplement: Supplementary file 1 [file jof-11-00595-s001.zip › jof-3804561-supplementary.pdf]

Table S1: Conidial germination rate and appressorial differentiation rate of *Beauveria bassiana* under different sulforaphane concentrations

Sulforaphane concentration (mg/mL)	Germination rate at 12 h (%)	Germination rate at 24h (%)	Appressorium formation rate at 24 h (%)	Germination rate at 36 h (%)	Appressorium formation rate at 36 h (%)	Germination rate at 48 h (%)	Appressorium formation rate at 48 h (%)	Germination rate at 72 h (%)	Appressorium formation rate at 72 h (%)
0	2.20±1.30 a	49.80±2.86 a	5.60±1.67 a	61.40±3.36 a	36.80±5.40 a	81.40±3.21 a	56.4±1.52 a	89.20±3.03 a	64.20±5.67 a
0.01	ND	32.60±3.21 b	1.40±1.14 b	56.60±2.41 ab	25.40±2.07 b	70.40±3.85 b	49.40±3.36 b	86.40±2.70 ab	52.60±4.88 b
0.02	ND	20.80±1.92 c	0.60±0.90 b	54.20±5.26 b	17.20±3.70 c	64.80±3.42 bc	41.00±2.00 c	85.60±1.52 b	46.20±3.03 c
0.05	ND	13.20±4.66 d	ND	25.80±3.03 c	15.20±3.35 c	62.00±4.12 cd	29.40±1.95 d	84.20±2.17 bc	38.60±3.85 d
0.10	ND	1.60±0.55 e	ND	19.20±3.70 d	1.00±1.00 d	58.00±4.90 d	24.00±2.74 e	81.40±1.67c	24.60±3.36 e

Note: Data in the table represent mean ± standard deviation. Different lowercase letters within the same column indicate statistically significant differences ($p \le 0.05$) in germination rates between treatments at the same germination time, while identical lowercase letters denote non-significant differences ($p > 0.05$). 'ND ' means not detected.

Table S2: Pathogenicity of different *Beauveria bassiana* attachment cell differentiation rates to test worms

Test Insect	Sulforaphane (mg/mL)	48h Attachment Cyst Formation Rate (%)	5d Cumulative Mortality Rate (%)	5d Adjusted mortality rate (%)	7d Cumulative mortality rate (%)	7d Adjusted mortality rate (%)	10d Cumulative mortality rate (%)	10d Adjusted mortality rate (%)	Cadaver rate (%)	Correlation coefficient R^2	Lethal Time 50% （d）	Regression equation
Opisina arenosella	0	62.20±3.35 a	43.00±4.47 a	38.04	83.00±4.47 a	80.90	96.00±4.18 a	95.50	89	0.947	5.4	Y = 13.11X - 20.81
	0.01	51.10±2.07 b	36.00±4.18 ab	28.57	75.00±5.70 bc	68.54	92.00±6.70 a	91.01	87	0.932	5.6	Y = 12.35X - 18.73
	0.02	42.50±1.95 c	30.00±3.53 bc	22.45	59.00±6.51 d	53.93	78.00±4.47 b	75.28	74	0.918	5.7	Y = 11.48X - 15.24
	0.05	28.00±2.70 d	26.00±2.23 c	19.57	49.00±4.18 e	42.70	71.00±4.18 b	67.42	63	0.901	5.9	Y = 10.52X - 12.46
	0.10	21.80±2.92 e	24.00±4.18 c	17.39	41.00±6.52 e	33.71	58.00±7.59 c	52.80	55	0.885	6.2	Y = 9.67X - 9.85
	Ck1	0.00±0.00 f	9.00±5.48 d	—	11.00±5.48 f	—	11.00±5.48 d	—	—	—	—	—
	Ck2	0.00±0.00 f	10.00±7.07 d	—	10.00±7.07 f	—	11.00±6.52 d	—	—	—	—	—
Bombyx mori	0	58.90±3.21 a	43.00±5.70 a	40.63	71.00±6.52 a	69.79	98.00±2.74 a	97.89	97	0.912	5.8	Y = 11.84X - 18.67
	0.01	49.40±5.40 b	36.00±6.52 b	33.33	68.00±5.70 ab	66.67	91.00±6.52 a	90.53	91	0.896	6.0	Y = 10.97X - 15.82
	0.02	40.20±3.03 c	25.00±3.54 c	21.88	62.00±5.70 b	60.42	80.00±5.00 b	78.95	80	0.879	6.3	Y = 9.83X - 12.39
	0.05	30.60±3.36 d	21.00±4.18 c	17.71	46.00±6.52 c	43.75	70.00±6.12 c	68.42	70	0.854	6.7	Y = 8.76X - 9.45

0.10	23.00±1.41 e	19.00±4.18 c	15.63	40.00±3.54 c	37.50	63.00±8.37 c	61.05	63	0.832	7.1	Y = 7.69X - 6.58
Ck1	0.00±0.00 f	4.00±4.18 d	—	4.00±4.18 d	—	5.00±6.12 d	—	—	—	—	—
Ck2	0.00±0.00 f	—	—	2.00±2.74 d	—	9.00±4.18 d	—	—	—	—	—

Note: Data in the table are presented as mean ± standard deviation. Different lowercase letters within the same column indicate significant differences among treatments ($p \leq 0.05$), while the same lowercase letters indicate no significant differences among treatments ($p > 0.05$)
